# *Leishmania* Infection Engages Non-Receptor Protein Kinases Differentially to Persist in Infected Hosts

**DOI:** 10.3389/fimmu.2016.00146

**Published:** 2016-04-18

**Authors:** Naixin Zhang, Peter E. Kima

**Affiliations:** ^1^Department of Microbiology and Cell Science, University of Florida, Gainesville, FL, USA

**Keywords:** *Leishmania*, protein kinases, Abl kinase, PKR, phagocytosis

## Abstract

Protein kinases play important roles in the regulation of cellular activities. In cells infected by pathogens, there is an increasing appreciation that dysregulated expression of protein kinases promotes the success of intracellular infections. In *Leishmania*-infected cells, expression and activation of protein kinases, such as the mitogen-activated protein kinases, kinases in the PI3-kinase signaling pathway, and kinases in the NF-κB-signaling pathway, are modulated in some manner. Several recent reviews have discussed our current understanding of the roles of these kinases in *Leishmania* infections. Apart from the kinases in the pathways enumerated above, there are other host cell protein kinases that are activated during the *Leishmania* infection of mammalian cells whose roles also appear to be significant. This review discusses recent observations on the Abl family of protein kinases and the protein kinase regulated by RNA in *Leishmania* infections.

## Introduction

Natural *Leishmania* infections are initiated by the deposition of promastigotes forms of *Leishmania* by sand flies at the site of their blood meal. Current understanding is that phagocytes, particularly neutrophils that are recruited earliest to the bite site become a sanctuary for the promastigotes (1). Once within mammalian cells, *Leishmania* commence to modify their gene expression profile, which culminates with their transformation into amastigote forms. By 24 h post-infection, the parasites are fully transformed into amastigote forms, which are the replicative form of *Leishmania* within mammalian cells and hosts. Some parasite species (*Leishmania tropica*, *Leishmania major*, *Leishmania mexicana*, and *Leishmania braziliensis*) replicate within inflammatory cells that are recruited to the bite site, which results in cutaneous lesions; other parasite species (*Leishmania donovani* and *Leishmania infantum*) traffic by still poorly understood mechanisms to the visceral organs where they take up residence and participate in the development of infected cell foci. Cells that are recruited to the site of infection are transformed by infection. Under conditions that continue to be investigated, infected cells are induced to release mediators some of which can promote parasite persistence, whereas others contribute to the control of infection. A few recent reviews have discussed our current understanding of the role of several protein kinases, such as the mitogen-activated protein kinases (MAPK), kinases in the PI3-kinase signaling pathway, and kinases in the NF-κB-signaling pathway (2–5), in the transformation of infected cells. In this review, the current understanding of the role of the Abl family of protein kinases that play an early role in transduction of signals will be discussed. Interactions with macrophage surface molecules, including the complement receptor, also plays a role in the activation of protein kinase regulated by RNA (PKR), which induces the release of IFNα, IL-27, and IL-10 among other cytokines that also modulate the host response to infection.

## Parasite Internalization

There has been long-standing interest in the molecular interactions that mediate *Leishmania* parasite entry into mammalian cells. Several phagocytic receptors, including the mannose receptor, scavenger receptor, complement receptors, and Fc receptors (6), have all been shown to be suitable internalization receptors of *Leishmania* parasites. However, in light of the fact that parasites in mammalian hosts are bathed in serum that contains opsonins including complement components and parasite specific antibodies, it is most likely that opsonin-dependent receptors are the preferred receptors that mediate parasite uptake. The critical importance of antibodies as opsonins for parasite internalization *in vivo* had been suggested by studies in animals that were genetically modified to be defective in circulating antibodies (7, 8). Those mice developed much smaller lesions as compared to wild-type mice when they were infected with *L. mexicana* parasites. Small lesions were proposed to be the result of not only reduced parasite uptake but also to be due to a skewed cytokine response (8). A few recent reports have revisited this topic and have explored the contributions of the opsonin-dependent receptors in mediating *Leishmania* parasite uptake by cells including neutrophils. We initially review the results of those studies to set the appropriate frame of reference for the discussion of the role of the Abl family kinases and PKR.

Numerous reports had assessed the role of the phagocytic receptor in the internalization of *Leishmania* parasites into macrophages. However, a recent study evaluated uptake of *L. donovani* parasites either *via* CR3 or the Fc receptors in the context of their effect on the maturation of the parasitophorous vacuole (PV). These studies were informed by Desjardins and Descoteaux (9) who had shown that upon internalization of *L. donovani* promastigote forms, the nascent PV undergoes a delay in its maturation. Acquisition of late endocytic pathway characteristics, characterized by the loss of early endosome autoantigen 1 (EEA1) and display of the lysosome associate membrane protein (LAMP-1) on the PV membrane, is delayed as compared to internalization of amastigotes that displays LAMP-1 within an hour of infection. Polando et al. (10) found that the phagocytic receptor that is engaged for parasite internalization affects PV maturation. Specifically, promastigote opsonization with C3-containing serum reduced the PV maturation delay by 2 h, whereas opsonization with *Leishmania* immune serum reduced the PV maturation delay by 3 h. In a follow paper, Ricardo-Carter et al. (11) showed that there are other biological consequences to the choice of entry receptor. Their contribution to the well-known phenomenon that *Leishmania*-infected cells do not secrete IL-12 and other inflammatory cytokines in response to lipopolysaccharide was that the underlying suppression mechanism is initiated upon the CR3-mediated uptake of parasites. They ruled out a role for signaling intermediates NFκB p65, MAPK, IRF-1, or IRF-8 in cytokine suppression induced by parasite uptake *via* CR3. Together, the studies described above are recent contributions to the long held appreciation of the role of the phagocytic receptor in parasite entry into mammalian cells.

As alluded to above, parasites are engulfed by phagocytes that recruited to the site of infection. Among these phagocytes are neutrophils that have been implicated in the “Trojan horse” infection strategy where they serve as a sanctuary for promastigote forms until promastigotes transform into amastigotes and are released or infected neutrophils in distress are engulfed by macrophages. Some of the dynamics of parasite uptake by neutrophils specifically with respect to the phagocytic receptors that mediate parasite entry were investigated by Soong and colleagues (12). When infections were performed in standard cell medium, Carlsen et al. found that although a greater proportion of promastigotes are killed after neutrophil internalization, both promastigote and amastigote forms of *Leishmania amazonensis* infect neutrophils comparably. Incubation of parasites in heat-inactivated serum obtained from *L. amazonensis*-infected mice significantly increased uptake by neutrophils of both promastigote and amastigote forms. Interestingly, tissue-derived amastigotes infected neutrophils at a slightly higher rate. Carlsen et al. then observed differences in the quality of neutrophil activation when infections were initiated by either promastigote or amastigote forms. Uptake of promastigote forms by neutrophils elicited reactive oxygen species (ROS) as well as the production of TNFα. By contrast, internalization of amastigotes by neutrophils resulted in the preferential release of IL-10 and ROS as well. Other consequences of this differential activation of neutrophils included the increase in the lifespan of infected neutrophils and their quality of death. Death by necrosis as compared to apoptotic death by neutrophils elicits dramatically different host responses (13). Differential engagement of the opsonin-dependent receptors by *Leishmania* parasites during their entry into neutrophils appears to result in similar outcomes as compared to macrophages.

## Role of Abl Family Kinases in Internalization of *Leishmania* Parasites

Of course, phagocytic receptors are associated with receptor linked kinases that transduce signals into the cell. No recent studies have evaluated the roles of these kinases in *Leishmania* infections. It should also be acknowledged that in addition to kinases, small GTPases, including Cdc42, Rac1, and Rho, play distinguishable roles in the uptake of particles as well as *Leishmania* parasites (14). That said, a report by Wetzel et al. (15) uncovered the important role of the Abl family of kinases in the uptake of *Leishmania* parasites *via* either the CR3 or the Fc receptor. The Abl family of protein kinases (first discovered as the oncogene in the Abelson leukemia virus) are non-receptor kinases that transduce signals from diverse extracellular stimuli that can result in cytoskeletal rearrangement during phagocytosis, cell to cell contact, and cell motility (16). They are composed of two members, Abl1 and Abl2 (Arg). Using bone marrow-derived macrophages obtained from mice genetically modified to be deficient in Abl1 (Abl^−/−^), Wetzel et al. found that there was an up to 42% reduction in the uptake of C3bi-opsonized *L. amazonensis* promastigotes as compared to the uptake of these parasites by cells from wild-type mice. Uptake of antibody-opsonized amastigotes was unaffected in Abl^−/−^ cells. They then showed that Imatinib (an Abl family kinase inhibitor) reduced C3bi-opsonized promastigotes uptake down to comparable levels as the Abl^−/−^. In addition, cells from mice that were genetically modified to be deficient in both Abl and Arg [because of embryonic lethality of double knockouts, this was achieved by using a conditional knockout strategy to inactivate the *abl* allele in an arg^−/−^ genetic background (henceforth called dbKO Abl/Arg)] were also shown to have reduced uptake C3bi-opsonized promastigotes. The role of the Abl kinase in mediating the entry of parasites *via* the CR3 receptor was supported by additional experiments in the mouse macrophage cell line RAW264.7. They showed that C3bi-opsonized promastigotes entry into RAW264.7 was significantly reduced when infections of these cells were performed in the presence of M1/70 (a CR3 blocking antibody) and not F16/32 (an FcR blocking antibody).

Parallel experiments were performed to evaluate the role of Arg kinases (other Abl family member) that has also been implicated in interactions with the cell cytoskeleton. Bone marrow-derived macrophages from mice that were engineered to be genetically deficient in Arg (Arg^−/−^) were found to take up C3bi-opsonized promastigotes at comparable levels with cells from wild-type mice. However, in these cells, uptake of IgG-opsonized amastigotes was reduced by 46%. Comparable reductions of opsonized amastigotes were observed in dbKO Abl/Arg. In addition, Imatinib also resulted in significantly reduced uptake of opsonized amastigotes. Although the uptake of C3bi-opsonized amastigotes was also reduced in Arg^−/−^ cells, these cells took up C3bi-coated beads as efficiently as did cells from wild-type mice. This latter observation with *Leishmania* amastigotes underscores the complexity of the internalization schemes employed by these parasites and their capacity to engage other receptors when needed.

The most remarkable part of the Wetzel et al. study was the *in vivo* experiments. It should be noted that in the *in vitro* studies, the experimental design called for short-term incubations of parasites with macrophages (20 min in the case of amastigote infections and 90 min in the case of promastigote infections), as these parasites can apparently employ alternate receptors to ensure their uptake. Remarkably, there was significant reduction in the course of infection in Arg^−/−^ mice. This was consistent with the *in vitro* observations that showed that Arg kinase played a significant role in the uptake of IgG-opsonized amastigotes. Further confirmation of the critical role of parasite uptake mediated by Arg kinase was obtained in dbKO Abl/Arg mice and in mice treated with Imatinib. Reduced lesion sizes in these mice corresponded with significant reductions in the parasite burden that was determined at the end of the experiment. Imatinib had been shown not to have a direct effect on parasite viability. The authors of the study concluded that the reduction in parasite burden and by consequence the limitation of the course of infection was due to the role of the Abl family kinases in the uptake of these parasites. Although Abl family kinases play important roles in T cell functions, T cell responses in the Abl family kinase knockout mice appeared as well as in the drug-treated mice appeared normal, which ruled out the possibility that alterations in the course of infections were the result of T cell abnormalities.

The Abl family of protein kinases might play a greater role in *Leishmania* infections than is presently appreciated. The observations with Imatinib suggest that this drug or a derivative could be useful in combination with other drugs that target the parasite to control *Leishmania* infections. Along these lines, the uptake of several bacterial pathogens, including *Shigella flexneri*, *Chlamydia trachomatis*, and *Mycobacterium* spp., has also been shown to utilize the Abl family of tyrosine kinases during their entry into cells (17–19). In infections by *Mycobacterium tuberculosis* and *Mycobacterium marinum*, Napier et al. showed that administration of Imatinib to infected mice reduced their bacterial load and associated pathology. Taken together, these studies lend support to the proposition that identification of cellular host genes that are exploited by pathogens could be targeted to control a significant group of pathogens (20).

## Protein Kinase Regulated by RNA

The PKR is an important antiviral kinase that promotes many cellular processes, including cytokine production. During some viral infections, the viral dsRNA binds to the N-terminal of PKR, which results in dimerization and autophosphorylation of PKRs. Once the PKR is activated, it phosphorylates and inhibits the eukaryotic translation initiation factor 2α (elf2α) to reduce overall translation levels of the host cell proteins (21). Besides the inhibition of translation, PKR can also activate NF-κB, which acts to increase the production of cytokines (such as IL-10 and type 1 interferons) from the host cell. PKR is also activated in bacterial infections; however, its role appears to be controversial. In some bacterial infections, PKR activation has been shown to be induced upon the interactions of their cell wall components with toll-like receptors (TLRs). PKRs in turn activate NF-κB signaling that culminates in the production of inflammatory cytokines, including TNF-α and IL-6 (22). However, as we discuss below, in some infections, activation of PKRs is disease promoting.

## Role of PKR in *L. amazonensis*

In several recent publications, Lopes and colleagues have provided evidence of an infection enhancing role of PKR in *L. amazonensis* infections. Pereira et al. (23) showed that *L. amazonensis* infection of cultured human or mouse macrophage cell lines induced the activation of PKR as monitored by the time-dependent increase in the phosphorylated form of PKR (pPKR). Supportive evidence of the increased activation of PKR by *L. amazonensis* infection was obtained by the observation of a time-dependent increase in the pelf2α. They then showed interestingly that infections performed in cells expressing a dominant negative mutant of PKR had lesser parasites than in controls, suggesting that pPKR induction augments the *L. amazonensis* parasite burden. Although activation of PKR by other stimuli such as poly(I:C) had been shown to be associated with induction of NO production, they found that *L. amazonensis* infection inhibited PKR-dependent NO production by a mechanism that involved aberrant induction of NF-κB; specifically, infection induced the translocation of the inhibitory p50/p50 homodimer into the nucleus. In a subsequent study by Barreto-de-Souza et al. (24), they showed that PKR augmentation of *L. amazonensis* infections occurs subsequent to its induction of IL-27, which is a cytokine that is structurally related to IL-12 (25). IL-27 that is produced in a time-dependent manner by *L. amazonensis* infection can be inhibited by expression of a dominant negative form of PKR. Addition of exogenous IL-27 enhanced the *L. amazonensis* load in infected cultures. Augmentation of *L. amazonensis* in cells was inhibitable by addition of anti-IL-27 to the infection cultures. Given that some reports have shown that IL-27 induces IL-10 production, which can in turn participate in the inhibition of *L. amazonensis* proliferation, Barreto-de-Souza et al. performed infections in the presence of exogenous IL-27 and antibodies to IL-10 receptor. Inhibition of IL-10 uptake by cells reduced the IL-27 enhancement of *L. amazonensis* infection.

These observations are consistent with studies that evaluated *M. tuberculosis* infections in mice that were deficient in PKR. PKR^−/−^ mice were found to contain fewer viable bacteria than wild-type mice after infection with Mtb (26). In addition, PKR^−/−^ mice exhibited less pulmonary pathology than wild-type mice. It was then shown that in the absence of PKR, infected cells were more prone to undergo apoptosis in response to Mtb infection and exhibited enhanced activation in response to IFN-γ. They reasoned that PKR promotes most likely induces a constitutive low level of IL-10 production that restrains macrophage activation and by so doing promotes pathogen persistence. Administration of a PKR inhibitor is therefore a plausible approach for pathogen control.

## Role of PKR in *L. major* Infections

An opposite role for PKR has been observed in *L. major* infections. Here, PKR activation is associated with increased *L. major* death within infected cells. This was observed acutely in infection studies in RAW264.7 macrophages where at 3 h post-infection the parasite burden in wild-type cells (RAW-Bla, transfected with empty plasmid) was comparable to the burden in cells expressing a dominant negative variant of PKR (RAW-DN-PKR) (27). However, at 24 h post-infection, the burden of *L. major* parasites in RAW-Bla cells was significantly less than in RAW-DN-PKR. Poly(I:C) had no effect on the survival of *L. major* in RAW-DN-PKR cells. It had previously been observed that unlike in infections with *L. amazonensis*, in which infection induces the translocation of the p50/p50 NF-κB homodimers into the nucleus, even upon poly(I:C) stimulation, *L. major* infection resulted in translocation of the stimulatory p65/p50 heterodimers. Evidently, *L. major* and *L. amazonensis* must express different infection promoting mechanisms. In light of previous studies that had characterized the inhibitor of parasite elastase (ISP) in *L. major* and that had shown that ISPs bind to neutrophil elastase (NE) on the surface of macrophages, the role of ISPs as potential regulators of macrophage responses to *L. major* was evaluated. Parasites that were deficient in ISPs (Δisp2/isp3) were derived and evaluated in infections of RAW-Bla and RAW-DN-PKR cells (27, 28). Interestingly, ISP-deficient cells were internalized more efficiently by RAW-Bla macrophages. They also survived better in RAW-DN-PKR macrophages, which suggested that PKR plays a role in reduced survival of *L. major* in macrophages. This observation was confirmed in infections of primary cells from PKR^−/−^ mice and wild-type mice (129Sv); *L. major* parasite burdens at 24 h post-infection were not different from those in cells for PKR^−/−^ mice. This was different from the observations in infections with *L. amazonensis* where a deficiency of PKR eliminated the augmentation of parasite burdens.

Additional studies to identify the cellular receptors that mediate the activation of PKR during *L. major* infections found that TLR2, TLR4, and CR3 most likely work in concert with neutrophil elastase to activate PKR. In light of differences in susceptibility of *L. major* to the induction of PKR, which is in contrast to *L. amazonensis* that is induced to replicate, Faria et al. (27) proposed that ISP characteristics of these species could be the significant difference. *L. major* parasites express higher levels of ISPs as compared to *L. amazonensis*. These ISPs then interact differentially with TLR2 and TLR4 that form a complex with neutrophil elastase and CR3. *L. amazonensis* appears to exhibit a greater preference for interactions with TLR2. Taken together, the parasite’s interactions with surface receptors, including CR3, set in motion the eventual activation of PKR that controls parasite replication, survival, as well as the release of critical cytokines by infected cells.

## Concluding Remarks

An understanding of how mammalian cells cope with or are transformed by *Leishmania* infection will necessitate a complete understanding of changes in activation of many host cell protein kinases. Parasite interactions with surface molecules on mammalian host cells initiates host cell responses that significantly influence the progress of the infection. The Abl family of protein kinases and also PKR are non-receptor protein kinases that play important roles in determining the outcome of *Leishmania* infections. Detailed studies of responses elicited to each *Leishmania* species underscore the differences between these parasites.

## Author Contributions

All authors listed, have made substantial, direct, and intellectual contribution to the work, and approved it for publication.

## Conflict of Interest Statement

The authors declare that the research was conducted in the absence of any commercial or financial relationships that could be construed as a potential conflict of interest.
